# Supplementary material for: Quantitative mapping of cerebrovascular reactivity amplitude and delay with breath-hold BOLD fMRI when end-tidal CO 2 quality is low
Source: Imaging Neurosci (Camb). 2025 Apr 8;3:imag_a_00536. doi: 10.1162/imag_a_00536 (PMC12319867; doi:10.1162/imag_a_00536)
Supplement: Supplementary Material [file imag_a_00536-supp.pdf]

Quantitative mapping of cerebrovascular reactivity amplitude and delay with breath-hold BOLD fMRI when end-tidal CO_2 quality is low

Rebecca G. Clements, Kristina M. Zvolanek, Neha A. Reddy, Kimberly J. Hemmerling, Roza G. Bayrak, Catie Chang, Molly G. Bright

Supplementary Material

Table S1. Task timings and number of datasets for each task in the in-house training dataset

**Task Number**	**1**	**2**	**3**	**4**
Number of datasets	112	58	20	55
Initial rest period duration (s)	20	15	0	0
Number of trials	7	5	6	10
Paced breathing duration (s)	24	24	24	[24, 30, 36] randomized with replacement
Breath hold duration (s)	18	18	[10, 12, 14, 16, 18, 20] randomized without replacement	[10, 11, 12, 13, 14, 15, 16, 17, 18, 19, 20] randomized with replacement, 10% chance that each breath hold is skipped and replaced with a rest period
Exhalation duration (s)	2	3	2	2

Recovery duration (s)	6	6	6	[6, 7, 8, 9, 10, 11, 12] randomized with replacement
End rest period duration (s)	30	15	0	0

Table S2. Correlations between measured $P_{ET}CO_2$ convolved with the HRF and RVT convolved with the HRF

**Datasets included**	**Average Fisher's Z between measured $P_{ET}CO_2$ and RVT**
Datasets with measured $P_{ET}CO_2$ timeseries containing only high-quality breath holds	1.04 ± 0.20
Datasets with measured $P_{ET}CO_2$ timeseries containing one or more low-quality breath holds	0.64 ± 0.27

Table S3. Error terms and hyperparameters for the 6 best performing models

Rank	Number of Layers	Number of Epochs	Loss Function	MAE (a.u.)	RMSE (a.u.)	RMSE at the peaks (a.u.)
1	12	20	MSE(true,predicted) +0.5*MSE($true_{peaks}$,$predicted_{peaks}$)	0.447	0.601	0.614
2	10	5	MSE(true,predicted)	0.409	0.555	0.674
3	8	5	MSE(true,predicted) +0.5*MSE($true_{peaks}$,$predicted_{peaks}$)	0.514	0.693	0.695
4	10	5	MSE(true,predicted) +0.5*MSE($true_{peaks}$,$predicted_{peaks}$)	0.473	0.669	0.705
5	10	15	MSE(true,predicted) +MSE($true_{peaks}$,$predicted_{peaks}$)	0.468	0.656	0.706

6	12	25	MSE(true,predicted)	0.383	0.515	0.710

Table S4. Effect sizes and *p*-values for 2-sided paired t-tests comparing the correlations, MAEs, and RMSEs of rescaled, predicted $P_{ET}CO_2$ and rescaled RVT. Asterisks indicate significant *p*-values

**Comparison**	***p*-value**	**Effect Size**
Correlation of predicted $P_{ET}CO_2$-1BH and RVT-1BH	0.0000*	0.6956
MAE of predicted $P_{ET}CO_2$-1BH and RVT-1BH	0.0000*	-1.1739
MAE of predicted $P_{ET}CO_2$-2BH and RVT-2BH	0.0000*	-1.1769
MAE of predicted $P_{ET}CO_2$-3BH and RVT-3BH	0.0000*	-1.1325
MAE of RVT-1BH and RVT-2BH	0.1645	0.1883
MAE of RVT-2BH and RVT-3BH	0.0036*	0.4064
MAE of predicted $P_{ET}CO_2$-1BH and predicted $P_{ET}CO_2$-2BH	0.0041*	0.4008
MAE of predicted $P_{ET}CO_2$-2BH and predicted $P_{ET}CO_2$-3BH	0.3043	0.1386
RMSE of predicted $P_{ET}CO_2$-1BH and RVT-1BH	0.0000*	-1.1585
RMSE of predicted $P_{ET}CO_2$-2BH and RVT-2BH	0.0000*	-1.1259
RMSE of predicted $P_{ET}CO_2$-3BH and RVT-3BH	0.0000*	-1.0714
RMSE of RVT-1BH and RVT-2BH	0.1887	0.1778
RMSE of RVT-2BH and RVT-3BH	0.0042*	0.3994
RMSE of predicted $P_{ET}CO_2$-1BH and predicted $P_{ET}CO_2$-2BH	0.0325	0.2932
RMSE of predicted $P_{ET}CO_2$-2BH and predicted $P_{ET}CO_2$-3BH	0.4831	0.0944

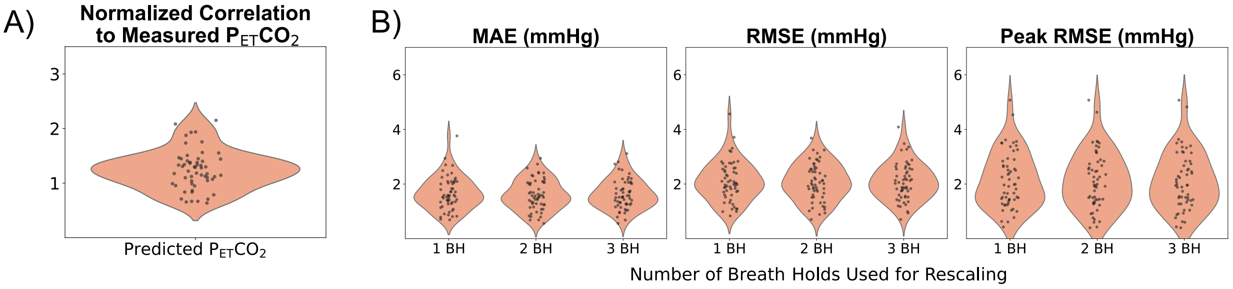

Figure S1. Overview of metrics comparing rescaled, predicted $P_{ET}CO_2$ to measured $P_{ET}CO_2$ in datasets in which all breath holds in the measured $P_{ET}CO_2$ timeseries were classified as high-quality. Metrics were calculated before predicted and measured $P_{ET}CO_2$ were convolved with the

hemodynamic response function. Both Fisher's Z values (A), which are not affected by rescaling, and error terms for each rescaling method (B) are shown.

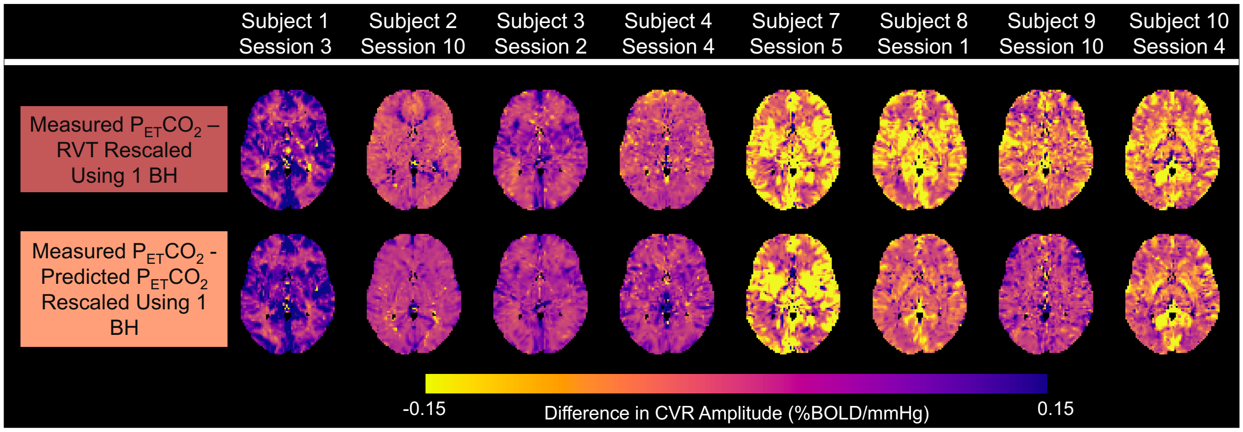

Figure S2. CVR amplitude difference maps for 8 subjects with measured $P_{ET}CO_2$ timeseries containing all high-quality breath holds (BHs). These maps show the differences between the ground-truth CVR amplitude values and the CVR amplitude values generated using the rescaled RVT regressor (top row), as well as the differences between the ground-truth CVR amplitude values and the CVR amplitude values generated using the rescaled, predicted $P_{ET}CO_2$ regressor (bottom row).

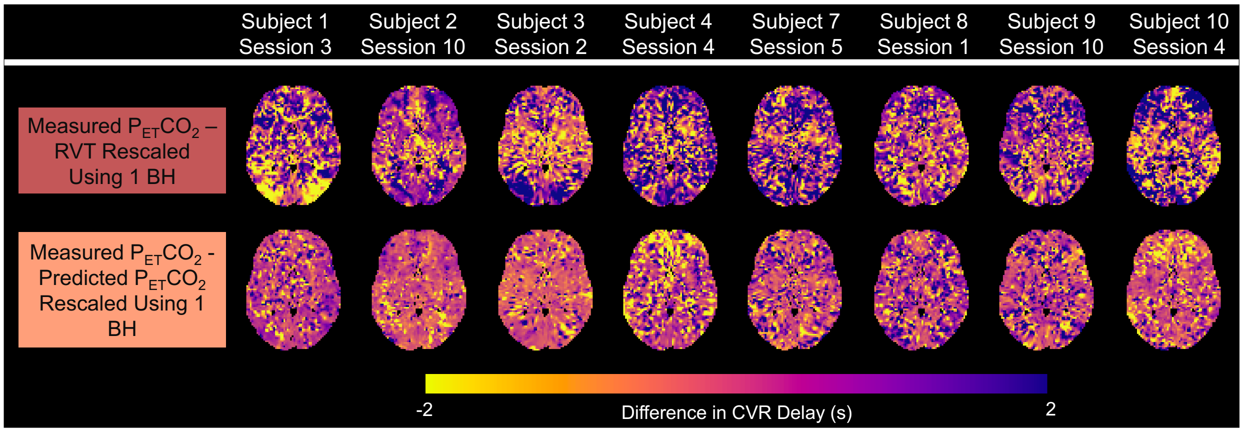

Figure S3. CVR delay difference maps for 8 subjects with measured $P_{ET}CO_2$ timeseries containing all high-quality breath holds (BHs). These maps show the differences between the ground-truth CVR delay values and the CVR delay values generated using the rescaled RVT regressor (top row), as well as the differences between the ground-truth CVR delay values and the CVR delay values generated using the rescaled, predicted $P_{ET}CO_2$ regressor (bottom row).

Table S5. Effect sizes and *p*-values for 2-sided paired t-tests comparing the correlations, MAEs, and RMSEs of CVR amplitude values in gray matter calculated using each regressor and rescaling method. Asterisks indicate significant *p*-values

**Comparison**	***p*-value**	**Effect Size**
Correlation of $CVR_{\text{predicted PETCO2-1BH}}$ and $CVR_{\text{RVT-1BH}}$	0.0000*	1.3234
MAE of $CVR_{\text{predicted PETCO2-1BH}}$ and $CVR_{\text{RVT-1BH}}$	0.0000*	-0.8604
MAE of $CVR_{\text{predicted PETCO2-2BH}}$ and $CVR_{\text{RVT-2BH}}$	0.0001*	-0.5809
MAE of $CVR_{\text{predicted PETCO2-3BH}}$ and $CVR_{\text{RVT-3BH}}$	0.0083	-0.3657
MAE of $CVR_{\text{RVT-1BH}}$ and $CVR_{\text{RVT-2BH}}$	0.0000*	0.5952
MAE of $CVR_{\text{RVT-2BH}}$ and $CVR_{\text{RVT-3BH}}$	0.0141	0.3386
MAE of $CVR_{\text{predicted PETCO2-1BH}}$ and $CVR_{\text{predicted PETCO2-2BH}}$	0.1862	0.1789
MAE of $CVR_{\text{predicted PETCO2-2BH}}$ and $CVR_{\text{predicted PETCO2-3BH}}$	0.1321	-0.2043
RMSE of $CVR_{\text{predicted PETCO2-1BH}}$ and $CVR_{\text{RVT-1BH}}$	0.0000*	-0.9413
RMSE of $CVR_{\text{predicted PETCO2-2BH}}$ and $CVR_{\text{RVT-2BH}}$	0.0000*	-0.7077
RMSE of $CVR_{\text{predicted PETCO2-3BH}}$ and $CVR_{\text{RVT-3BH}}$	0.0004*	-0.5063
RMSE of $CVR_{\text{RVT-1BH}}$ and $CVR_{\text{RVT-2BH}}$	0.0000*	0.5894
RMSE of $CVR_{\text{RVT-2BH}}$ and $CVR_{\text{RVT-3BH}}$	0.0090	0.3619
RMSE of $CVR_{\text{predicted PETCO2-1BH}}$ and $CVR_{\text{predicted PETCO2-2BH}}$	0.1165	0.2131
RMSE of $CVR_{\text{predicted PETCO2-2BH}}$ and $CVR_{\text{predicted PETCO2-3BH}}$	0.2013	-0.1728

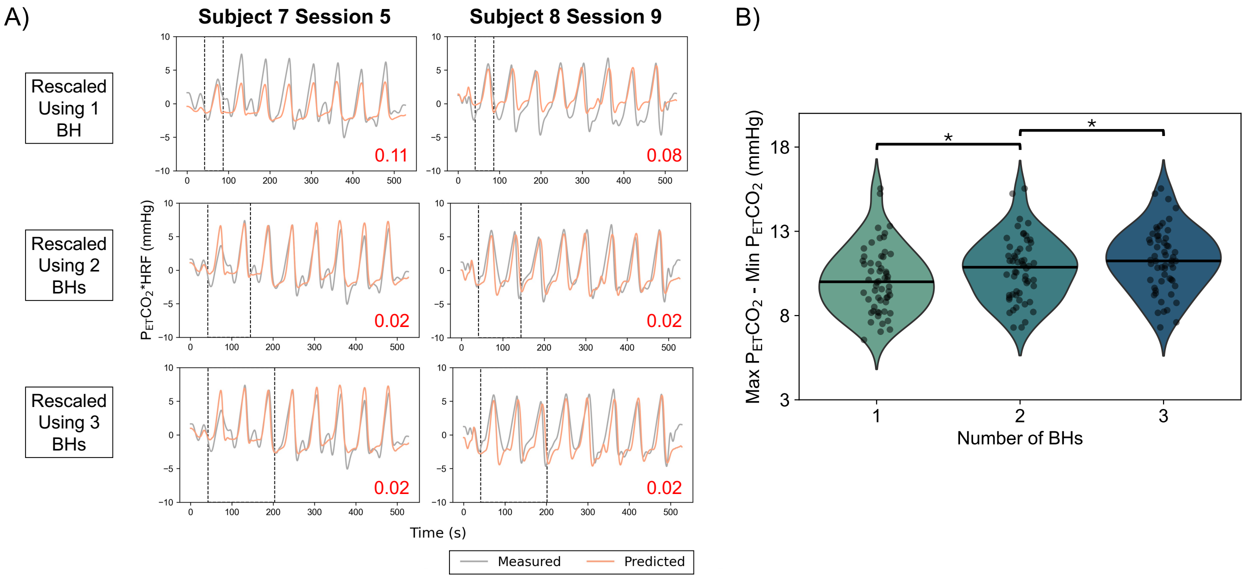

Figure S4. Section A shows example measured and rescaled, predicted $P_{ET}CO_2$ regressors for 2 example sessions in which rescaling using 1 breath hold (BH) was inaccurate and resulted in much higher CVR amplitude errors than rescaling using 2 or 3 BHs. Dashed lines indicate the section of measured $P_{ET}CO_2$ used for rescaling. For each rescaling method, the MAE (%BOLD/mmHg) of CVR amplitude in gray matter, calculated using the rescaled, predicted $P_{ET}CO_2$ CVR amplitude relative to the measured $P_{ET}CO_2$ CVR amplitude, is shown in red in the bottom right corner. For these example sessions, rescaling using more breath holds decreased the error of CVR amplitude. Section B shows the range of $P_{ET}CO_2$ values used for rescaling using 1, 2, and 3 breath holds for each session with all high-quality breath holds. Asterisks indicate significant differences between groups, determined using 2-sided paired *t*-tests (significance threshold $p<0.05$, with Bonferroni correction).
